# Associations between outdoor temperature and markers of inflammation: a cohort study

**DOI:** 10.1186/1476-069X-9-42

**Published:** 2010-07-23

**Authors:** Jaana I Halonen, Antonella Zanobetti, David Sparrow, Pantel S Vokonas, Joel Schwartz

**Affiliations:** 1 Department of Environmental Health, Harvard School of Public Health, Boston, MA, USA; 2 School of Public Health and Clinical Nutrition, University of Kuopio, Kuopio, Finland; 3 VA Normative Aging Study, Veterans Affairs Boston Healthcare System, Boston, MA, USA; 4 The Department of Medicine, Boston University School of Medicine, Boston, MA, USA; 5 Harvard School of Public Health, Landmark Center West, Room 415 E, 401 Park Drive, Boston, MA 02215, USA

## Abstract

**Background:**

Associations between ambient temperature and cardiovascular mortality are well established. This study investigated whether inflammation could be part of the mechanism leading to temperature-related cardiovascular deaths.

**Methods:**

The study population consisted of a cohort of 673 men with mean age of 74.6 years, living in the greater Boston area. They were seen for examination roughly every 4 years, and blood samples for inflammation marker analyses were drawn in 2000-2008 (total of 1254 visits). We used a mixed effects model to estimate the associations between ambient temperature and a variety of inflammation markers (C-reactive protein, white blood cell count, soluble Vascular Cell Adhesion Molecule-1, soluble Intercellular Adhesion Molecule-1, tumor necrosis factor alpha, and interleukins -1β, -6 and -8). Random intercept for each subject and several possible confounders, including combustion-related air pollution and ozone, were used in the models.

**Results:**

We found a 0 to 1 day lagged and up to 4 weeks cumulative responses in C-reactive protein in association with temperature. We observed a 24.9% increase [95% Confidence interval (CI): 7.36, 45.2] in C-reactive protein for a 5°C decrease in the 4 weeks' moving average of temperature. We observed similar associations also between temperature and soluble Intercellular Adhesion Molecule-1 (4.52%, 95% CI: 1.05, 8.10, over 4 weeks' moving average), and between temperature and soluble Vascular Cell Adhesion Molecule-1 (6.60%, 95% CI: 1.31, 12.2 over 4 weeks' moving average). Penalized spline models showed no deviation from linearity. There were no associations between temperature and other inflammation markers.

**Conclusions:**

Cumulative exposure to decreased temperature is associated with an increase in inflammation marker levels among elderly men. This suggests that inflammation markers are part of intermediate processes, which may lead to cold-, but not heat-, related cardiovascular deaths.

## Background

The association between cardio-respiratory mortality and ambient temperature is well known. More recently, associations have been found especially between mortality and heat wave episodes [1-3], but also between mortality and exposure to cold [4-6]. Additional adverse health effects by temperature may occur due to the current increase in the temperature variability caused by global warming [7,8]. However, the mechanisms underlying the temperature-related deaths, and the possibility to use markers of inflammation as biomarkers of these mechanisms, are still poorly understood.

Inflammation and vascular dysfunction are among the proposed mechanisms advancing cardiovascular events and atherosclerosis. There is evidence that increased levels of, for example, white blood cell count, a marker for inflammation, is a predictor of coronary events [9]. Increases in the levels of more commonly studied inflammation markers such as C-reactive protein (CRP) and interleukin 6 (IL-6) have been linked to increased risk of cardiovascular events [10-12]. Recent findings also suggest that IL-6 and CRP are more strongly associated with fatal rather than nonfatal cardiovascular events [13]. However, the causality of the association between CRP and cardiovascular diseases is not undisputed as elevated CRP has also been suggested to be a marker of the extent of atherosclerosis or a marker for the inflammation activity rather than cause of cardiovascular disease [14].

Interleukin-6 and 1-beta (IL-1β) belong to the group of acute phase proteins and are released into circulation as a response to infections or injuries. Other commonly known acute phase proteins are interleukin 8 (IL-8), and tumor necrosis factor alpha (TNFα) [15]. In a review of inflammatory markers of cardiovascular diseases, Ramos and co-authors [10] suggested that IL-6 and TNFα may be more useful than CRP in predicting cardiac risks among the elderly. Coronary heart disease and congestive heart failure have been linked to TNFα levels among the elderly [16], and the cascade leading to development and destabilization of atherosclerotic plaques to IL-1β [17]. Other markers of inflammation that may predict cardiovascular disease are soluble forms of intercellular adhesion molecule-1 (sICAM-1) and vascular cell adhesion molecule-1 (sVCAM-1) [18], which are interrelated markers of inflammation and endothelial function. However, the potential for outdoor temperature to affect the low-grade systemic inflammation as part of the mechanism leading to cardiovascular mortality has scarcely been studied. To our knowledge, only two studies from Europe have evaluated these associations, using selected inflammatory markers, and they reported inconsistent findings [19,20]. In addition, in these studies limited data for the summer time temperatures was available.

We therefore studied the possible associations between temperature and inflammation markers using data for the whole year, but controlling for season. To do so, we evaluated changes in the levels of high sensitive (hs) CRP, IL-1β, IL-6, IL-8, TNFα, sVCAM-1, sICAM-1 and white blood cell count (WBC) in association with a 5°C decrease in mean ambient temperature.

## Methods

### Study population

The study population consisted of the Normative Aging Study cohort of men living in the Greater Boston area. The cohort was established by the Veterans Administration in 1963 when men aged 21-80 years, confirmed to be free of known chronic medical conditions, were enrolled [21]. Subjects were asked to return for physical examinations and to complete questionnaires on factors possibly affecting health every 3-5 years. Covariate data (weight, height, medication use etc.) was updated at each visit, and blood samples for the markers of inflammation were drawn during each visit between June 2000 and August 2008 for participants (N = 708) still presenting for examination. All subjects provided written informed consent prior to attending the research, and this investigation has been approved by the Institutional Review Boards of Harvard School of Public Health and Normative Aging Study, Veterans Affairs Boston Healthcare System.

### Markers of inflammation

Of the biomarkers used in this study hs CRP, sVCAM-1 and sICAM-1 were assayed at Dr. Nader Rifai's laboratory at Children's Hospital, Boston, MA. High sensitive CRP concentrations in the serum were determined using an immunoturbidimetric assay on the Hitachi 917 analyzer (Roche Diagnostics, Indianapolis, IN), using reagents and calibrators from Denka Seiken (Niigata, Japan) [22]. Soluble ICAM-1 and soluble VCAM-1 levels in plasma were measured by using the Enzyme-Linked Immunosorbent Assay (ELISA) method (R & D Systems, Minneapolis, MN), with a sensitivity of 0.35 ng/ml for sICAM-1, and 2.0 ng/ml for sVCAM-1.

Interleukins -1β, -6, and -8, TNFα and WBC were analyzed at Molecular Epidemiology Laboratory of Harvard School of Public Health. Interleukins in serum samples were determined by Chemiluminescent ELISA as previously described [23]. TNFα was analyzed with Luminex xMAP multiplexing technology using the Luminex^® ^100/200™ System (Luminex, Austin, TX). This technology allows for sensitive and accurate measurement of up to 100 analytes simultaneously in 25 μl of serum. All samples from each participant were analyzed in duplicate and in one batch to avoid between-batch analytical variation. White blood cell count was determined using a standard automated electrical impedance hematology analyzer (Coulter Counter Model S-Plus Series - Coulter Electronics). The performance of the assays was monitored with standard quality control procedures including the analysis of blinded pooled samples.

From the data, we excluded visits with hs CRP levels that were greater than 10 mg/l because those are generally considered as clinically significant infectious inflammatory states [24]. After excluding these values and all missing data, we had 673 participants with 1 to 4 clinic visits resulting in total of 1254 visits (685 and 569 during warm (May-September) and cold (October-April) season, respectively).

### Meteorological and Air pollution Measurements

Ambient temperature (°C), relative humidity (%) and barometric pressure (millibar) measurements were derived from the Boston Logan Airport weather station located 8 km from the physical examination site. Continuous black carbon measurements were performed using an aethalometer (Magee Scientific, Berkeley, California) at the Harvard School of Public Health monitoring site, 1 km from the examination site. Ambient ozone was measured continuously at four monitoring sites in the Greater Boston area. Monitors were located in the cities of Boston, Chelsea, Lynn and Waltham and all of them conformed to US Environmental Protection Agency (EPA) standards. In the analyses we used the average of the four measurements.

### Statistical Analyses

All biomarker levels were log_10_-transformed to approximate normal distribution and to stabilize variance. We used mixed effects models to examine possible associations between ambient temperature and the levels of biomarkers. This method takes into account the weighted effect of repeated measurements. We do note that the estimated random intercepts for subjects with only one measurement are noisily estimated; however, the inferences for the fixed effects, such as temperatures, are appropriate in these models. Even though this method is used especially for dependent measures, mixed models do not require repeated measures for all subjects.

We adjusted models for *a priori *chosen known or plausible confounders including specific personal and temporal characteristics. All models included random effects for subjects and fixed effects for body mass index (BMI, as a continuous variable), age, cigarette smoking (never/former/current), alcohol consumption (≥2 drinks/day: yes/no), use of any antihypertensive medication and statins (yes/no), diabetes (yes/no), hypertension (yes/no), fasting blood glucose level (1: < 110 mg/dl, 2: > 110 or < 126 mg/dl, 3: > 126 mg/dl), race and years of education. Models included also indicator variables for season (warm/cold), weekday, and sine and cosine terms for day of year to capture seasonality more effectively. Additionally, the 24-hour means of relative humidity and barometric pressure were considered as possible confounders. The analyses were performed with statistical software R 2.10.1. using the linear and non-linear mixed effects models library (nlme) [25].

The effects of temperature on morbidity and mortality have been seen over lag periods up to 7-25 days [26,27], but more strongly at shorter lags. Therefore we chose *a priori *to analyze single-lag days from 0 to 14, and the moving averages of 7, 14, 21 and 28 days. Moving average of 7 days included lag days 0 to 6 prior to the clinic visit and is referred as "1 week" in tables and figures, moving average of 14 days included lag days 0 to 13 and is referred as "2 weeks", etc.

As secondary analyses, we studied interaction between temperature and obesity (BMI > 30 at the first visit in this dataset) to uncover possible effect modification, because obesity itself is considered as a low-grade inflammation state [24]. Because diabetes was previously found to be an effect modifier of the association between temperature and inflammation in coronary heart disease patients [20], we studied if the same was true in our cohort. Furthermore, effect modification by age has been found in temperature-health associations, and therefore we studied also interactions between temperature and age. We studied the possible confounding by combustion-related air pollution by adjusting the models for black carbon, because increases in the levels of CRP [28,29], IL-6 [30,31] and sVCAM-1 [32] have been independently associated with increases in the levels of combustion-related pollutants. To do so, we used the same lag for black carbon as for temperature in the models. The possible confounding effect of ozone was studied similarly, as exposure to ozone has also been associated with inflammation [33].

As sensitivity analyses, we inspected the linearity of the associations between temperature and biomarkers using plots created by generalized additive mixed model (gamm) with the mgcv library [34] in R, and we excluded the 2.5% of the hottest and the coldest days from the data. Cut offs for the hottest temperatures were: 27.9°C for lags 0 and 1, and 23.8 - 24.9°C for the moving averages, and for the coldest temperatures: -4.8°C for lags 0 and 1, and -1.97 - -4.56°C for the moving averages. Finally, we performed analyses for lag days 0 and 1 including a covariate for the indoor temperature of the examination room using a smaller data set (1087 clinical visits for 615 subjects). Less data was available because indoor temperature was measured for a shorter time period than the inflammation markers.

## Results

The mean (sd) ambient temperature over the study period was 12.4°C (9.0), and the indoor temperature for the measured period was 23.8°C (1.6). We used only one temperature measurement site for our analyses. However, the correlation between temperatures at Boston Logan Airport and T.F. Green Airport (Warwick, RI), that is a closer site to the study subjects living the furthest from Boston, was 0.96 (mean distance of the address of participants from airport was 18 km). This suggests that there is little variation in the temperature over the study area. Summary statistics of the environmental variables is in Table 1.

**Table 1:** Summary statistics of the environmental variables

Variable	Min	Mean (sd)	Max
Ambient Temperature (°C)	-13.9	12.4 (9.0)	31.7
Relative Humidity (%)	26.6	67.9 (15.7)	99.3
Barometric pressure (mbar)	983.6	1016.0 (7.9)	1036.0
Black Carbon (μg/m^3^)	0.12	0.84 (0.44)	2.7
O_3 _(ppb)	3.37	23.6 (12.3)	85.1

The age of the study subjects was within the range of 55-100 years, and 26.1% of them were obese (n = 176), with body mass index > 30 at the first visit. Of the 673 subjects, 245 had only one clinic visit, 276 had two visits, 151 had three visits, and one had four visits. More complete summarization of the study subjects' characteristics is provided in Table 2. Spearman rank correlations between temperature, inflammation markers and the pollutants are provided in the additional file 1. All results are presented as a % change in the biomarker level for a 5°C decrease in temperature with 95% Confidence Interval (CI). The decrease of five degrees was chosen because this is within the range of daily temperature variation in the study area.

**Table 2:** Descriptive statistics of the individual characteristics of the study subjects

Variable	Mean (sd)
C-reactive protein (mg/l)	2.3 (2.1)
White blood cell count (1000/mm^3^)	6.4 (3.0)
Interleukin 1 beta (pg/ml)	33.0 (123.9)
Interleukin 6 (pg/ml)	172.5 (351.4)
Interleukin 8 (pg/ml)	75.9 (84.2)
Soluble Vascular Cell Adhesion Molecule (ng/ml)	1066 (400.0)
Soluble Intercellular Adhesion Molecule (ng/ml)	299.0 (77.2)
Tumor necrosis factor alpha (pg/ml)	60.0 (199.2)
Age (years)	74.6 (6.6)
Body mass index (kg/m^2^)	28.1 (4.2)
Education (years)	14.6 (2.8)
	
	N subjects (%)
Obese (Body mass index > 30)	176 (26.1)
Use of statins	378 (56.2)
Use of any antihypertensive medication	483 (71.8)
Diabetes	114 (17.0)
Hypertension	542 (80.5)
Race	
Non-Hispanic white	649 (96.4)
Non-Hispanic black	12 (1.8)
Hispanic white	9 (1.3)
Hispanic black	3 (0.4)
	
Weekday of the visit	N observations (%)
Tuesday	363 (28.9)
Wednesday	741 (59.1)
Thursday	150 (11.9)
Smoking status	
Never	368 (29.3)
Former	840 (67.0)
Current	46 (0.4)
Fasting blood glucose	
< 110 mg/dl	906 (72.2)
> 110 or < 126 mg/dl	202 (16.1)
> 126 mg/dl	146 (11.6)
Alcohol intake(≥ 2 drinks/day)	236 (18.8)

**Figure 1:**
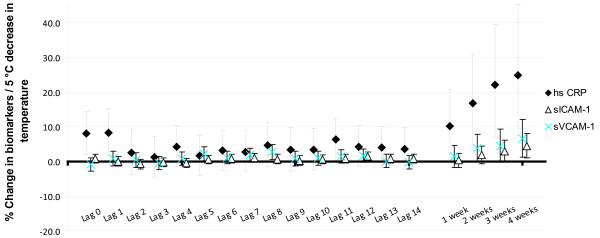
**The % change in inflammation markers for a 5°C decrease in temperature**. The % change (95% confidence intervals) in high sensitive C-reactive protein, soluble intercellular adhesion molecule-1, and soluble vascular cell adhesion molecule-1 in association with a 5°C decrease in ambient temperature.

We found positive associations between decrease in ambient temperature and the levels of hs CRP, sICAM-1 and sVCAM-1 throughout all investigated lags (Figure 1). Significant associations between temperature and hs CRP were observed with acute, 0 to 1 day, and cumulative lags (Table 3). To study whether the acute and cumulative effects were independent of each other, we run models including lag 0 or lag 1 with the 4 weeks' moving average for the temperature. The correlation between temperature on the current day and the 4 week's moving average was 0.88, and between lag day 1 and 4 week's moving average 0.90. All effect estimates were slightly reduced in the two-lag models, possibly due to rather high correlation between temperatures. However, the associations at lags 0 and 1 were still borderline significant and the association over the 4 weeks' moving average remained significant (20.0%, 95% CI: 2.65, 40.2, when analyzed with lag 0). Associations between temperature and adhesion molecules sVCAM-1 and sICAM-1 were the strongest over the cumulative lags (Table 3). We did not find significant associations for the other inflammation markers (See additional files 2 and 3).

**Table 3:** The % change in the levels of hs CRP, soluble VCAM-1, and soluble ICAM-1 for a 5°C decrease in ambient temperature

	**Original model **^**a**^		**Adjusted for BC **^**b**^		**Adjusted for O**_**3 **_^**c**^	
	**% Change**	**95% CI**	**% Change**	**95% CI**	**% Change**	**95% CI**

hs C-reactive protein						
Lag 0	8.02 *	1.93, 14.8	7.03 *	0.60, 13.9	9.39 *	2.97, 16.2
Lag 1	8.18 *	1.57, 15.2	10.0 *	3.09, 17.4	9.26 *	2.63, 16.6
1 week	10.2 *	0.51, 20.8	10.1	-0.12, 21.3	12.6 *	2.41, 23.8
2 weeks	16.7 *	4.05, 30.8	15.0 *	2.06, 29.6	19.2 *	6.11, 34.0
3 weeks	22.1 *	6.89, 39.6	19.7 *	4.12, 37.6	23.3 *	7.72, 41.0
4 weeks	24.9 *	7.36, 45.2	23.4 *	5.53, 44.2	26.3 *	8.50, 47.0
Soluble vascular cell adhesion molecule-1						
Lag 0	-0.94	-2.68, 1.02	0.41	-1.66, 2.52	-0.48	-2.49, 1.57
Lag 1	0.77	-1.36, 2.94	1.57	-0.62, 3.82	0.63	-1.56, 2.88
1 week	1.44	-1.66, 4.64	2.49	-0.82, 5.91	0.64	-2.52, 3.91
2 weeks	3.80	-0.13, 7.89	4.96 *	0.18, 9.28	2.82	-1.15, 6.95
3 weeks	4.41	-0.19, 9.22	5.70 *	0.84, 10.8	3.71	-0.89, 8.53
4 weeks	6.60 *	1.31, 12.2	8.04 *	2.51, 13.9	5.83 *	0.56, 11.4
Soluble intercellular adhesion molecule-1						
Lag 0	0.79	-0.51, 2.10	0.71	-0.68, 2.11	1.31	-0.01, 2.71
Lag 1	0.02	-1.39, 1.44	-0.08	-1.53, 1.39	0.32	-1.12, 1.79
1 week	0.31	-1.74, 2.40	0.91	-1.26, 3.13	0.22	-1.88, 2.36
2 weeks	1.82	-0.76, 4.47	2.95 *	0.23, 5.74	1.41	-1.21, 4.10
3 weeks	2.91	-0.12, 6.04	4.64 *	1.42, 7.92	2.59	-0.45, 5.72
4 weeks	4.52 *	1.05, 8.10	6.42 *	2.79, 10.2	4.23 *	0.76, 7.82

* *P*-value < 0.05

^a ^Model adjusted for relative humidity, barometric pressure, season, time trend, weekday, age, smoking, use of any anti-hypertensive medication and statins, body mass index, hypertension, education, diabetes, alcohol use (≥ 2 drinks/day), and race

^b ^Model adjusted for black carbon, relative humidity, barometric pressure, season, time trend, weekday, age, smoking, use of any antihypertensive medication and statins, body mass index, hypertension, education, diabetes, alcohol use (≥ 2 drinks/day), and race

^c ^Model adjusted for ozone, relative humidity, barometric pressure, season, time trend, weekday, age, smoking, use of any antihypertensive medication and statins, body mass index, hypertension, education, diabetes, alcohol use (≥ 2 drinks/day), and race

When we adjusted the model of hs CRP for black carbon we observed little changes in the associations between temperature and hs CRP, but adjusting for O_3 _made the associations slightly stronger (Table 3). The associations between temperature and adhesion molecules became slightly stronger after adjustment for black carbon, but remained similar to original model after adjustment for O_3 _(Table 3). We found no interactions between temperature and obesity, diabetes or age.

Adjusting the models for room temperature had minor effects on the effect estimates for 0 and 1-day lags (Additional file 4). As a test of the linearity assumption, we fit penalized splines for temperature using a gamm when the software chose the optimized degrees of freedom for temperature using generalized cross validation. This was 1 degree of freedom for hs CRP and sICAM-1 when using the moving average of temperature over 4 weeks, suggesting that the effects were linear. When we excluded the extreme temperatures (32 cold and 22 hot days excluded, total of 1190 observations used in the analysis) from the data, the observed associations at the cumulative lags became slightly stronger (Table 4).

**Table 4:** The % change in the levels of hs CRP, soluble VCAM-1, and soluble ICAM-1 for a 5°C decrease in ambient temperature excluding the extreme temperatures

	2.5% Hottest and coldest excluded	
	**% Change**	**95% CI**

C-reactive protein		
Lag 0	7.16 *	0.33, 14.5
Lag 1	4.41	-2.61, 11.9
1 week	1.69	-8.29, 12.7
2 weeks	8.26	-4.84, 23.1
3 weeks	20.5 *	3.51, 40.2
4 weeks	29.2 *	2.93, 45.1
Soluble vascular cell adhesion molecule-1		
Lag 0	-1.00	-3.16, 1.20
Lag 1	0.92	-1.41, 3.32
1 week	1.37	-1.14, 7.82
2 weeks	3.24	-2.45, 8.99
3 weeks	3.35	-1.77, 8.85
4 weeks	6.70 *	0.75, 13.0
Soluble intercellular adhesion molecule-1		
Lag 0	0.55	-0.92, 2.03
Lag 1	0.41	-1.14, 1.98
1 week	0.77	-1.53, 3.12
2 weeks	1.72	-1.17, 4.70
3 weeks	3.84 *	0.45, 7.34
4 weeks	6.01 *	2.10, 10.1

* *P*-value < 0.05

^a ^Model adjusted for relative humidity, barometric pressure, season, time trend, weekday, age, smoking, use of any anti-hypertensive medication and statins, body mass index, hypertension, education, diabetes, alcohol use (≥ 2 drinks/day), and race

## Discussion

In the present study, we found an increase in the levels of three inflammation makers; hs CRP, sICAM-1, and sVCAM-1, in association with a 5°C decrease in ambient temperature. We found no evidence of associations between temperature and other markers of inflammation. These findings suggest that some of the inflammation markers could be used as biomarkers of the intermediate processes leading to cardiovascular mortality related to exposure to decreasing temperatures.

We found acute and cumulative effects of temperature on hs CPR levels, the most commonly used marker for low-grade inflammation for predicting changes in cardiovascular health. In both cases, the levels of hs CRP increased linearly in association with decreasing temperature, and the effects were independent of each other. The acute effects are biologically plausible since serum levels of CRP can increase rapidly, reaching the level of 5 mg/l in six hours [35]. The cumulative effects can indicate an ongoing inflammation triggered by repeated exposures to decreasing temperatures. Cumulative effects may also be present because the recovery from illnesses and inflammation among the elderly may be slow. When the extreme temperatures were excluded, the associations became slightly stronger for the cumulative lag, providing further support for our findings. The direction of the association we found is consistent with the findings of a multi-city panel study of myocardial infarction survivors in Europe, where decrease in temperature predicted increase in the levels of CRP [19]. The data in that study; however, was mainly from the cold season including only one city with measurements also for the whole summer, which may have resulted in underestimation of the effects of warmer temperatures. Contradictory findings for winter season in Germany have been recently reported by Hampel et al. [20], who found a decrease in hs CPR levels in association with decrease in temperature among men with coronary heart disease. The contradictory in the findings may be related to different study populations, as Hampel et al. studied individuals diagnosed with cardiovascular or pulmonary disease.

The effects of temperature on adhesion molecule levels have been evaluated only once before, and only for the cold season in the study by Hampel et al. [20]. The authors observed a 5-day cumulative increase of 4.6% (95% CI, 0.2, 9.1%) in sICAM-1 for a 10°C decrease in temperature among men with coronary heart disease. The direction of the association is again consistent with our results, but the time lag in which the association was most prominent in the current study was longer; 3 to 4 weeks, while the association over the 7-day moving average was positive but not significant. We found similar associations between temperature and sVCAM-1 to those between temperature and sICAM-1. Similarity of these findings was expected due to moderate correlation (0.40) between sICAM-1 and sVCAM-1, even though higher short-term reactivity of ICAM-1 than VCAM-1 after exposure to exogenous factors has been reported in cell studies [36].

Current and previous [19] findings suggest a linear association between temperature and inflammatory markers, which is only partly consistent with the proposed U- and V-shaped associations between temperature and cardiovascular mortality [5,37,38]. Results from the study by Schneider and co-authors [19] seem to reflect the left part of the U- or V-shaped association between temperature and mortality, and therefore suggesting that inflammation may have a role in cold-related cardiovascular deaths. Our finding of an increase in the levels of inflammation markers in association with decreasing temperature, controlling for season, also suggests that inflammation may be part of the intermediate processes leading to cardiovascular mortality related to decreasing, but not increasing, temperature. While not consistent with the majority of the mortality studies, our findings are in line with recent findings where an inverse association between temperature and myocardial infarction mortality in summer was reported [6]. Our results could also reflect the finding that in the U.S. there has been a decline in heat-related cardiovascular deaths during recent years, whereas the effects of cold temperature have persisted [39]. There is also similarity in the time lags in which the associations are observed for biomarkers and mortality. In this study, the associations were mainly 3 to 4 weeks cumulative, when associations between low temperature and mortality have been reported to occur up to 14 to 25 days after exposure [26,27]. Nevertheless, more research using different study populations and at different locations is needed to confirm our findings.

An association between CRP and particles from traffic has recently been reported [28], but in our study, black carbon, a marker of combustion-related air pollution, did not confound the association between temperature and hs CRP. We observed minor confounding by black carbon, with additive effects, when we analyzed associations between temperature and adhesion molecules. A previous study on our study cohort found an association between black carbon and sVCAM-1 with 2 days delay [32], and the current results suggest that more cumulative associations between black carbon and adhesion molecules may also exist. Ozone is a reactive gas that has been linked to increase in CRP in one study [33], but not in others [40,41]. We found minor confounding effect by ozone, which lead to strengthening of the association between temperature and hs CRP. However, ozone did not confound the associations between temperature and adhesion molecules. We did not observe effect modification by obesity in this study, even though obesity itself may lead to low-grade inflammatory state, because adipose tissue is able to produce inflammatory cytokines [42]. In addition, we did not find effect modification by diabetes, as was reported in a previous study [20].

We studied a cohort of elderly individuals, who have been found to be a vulnerable subgroup with regard to low temperatures [43]. Because climate change introduces greater variability in temperature, it would be reasonable to take proactive actions against cold and sudden changes in weather. For example, adequate clothing, and good insulation and proper indoor heating of the residences of the elderly should be provided. Doctors could also recommend their cardiac patients and the elderly to follow weather forecasts in order to get prepared for changes in temperature.

Our study was subject to some limitations. The study population was homogenous including only elderly men living within a geographically limited area, so one should be cautious about making extrapolations of these results to other population groups. We did not have data on the time-activity patterns of the study subjects considering time spent indoors and outdoors, but we did attempt to control for the effect of the temperature in the examination room, which seemed to have little effect on the results found for acute lags. The previous studies addressed the same problem of exposure assessment, but it seems plausible that because cardiovascular mortality is associated with changes in outdoor temperature, the same can be true for inflammation markers [19,20]. Additionally, we had outdoor temperature measurements from only one monitoring site, which may not perfectly mirror the exposure of the whole study population; however, as the correlation between temperature measurements at two airports 100 km apart was high, the variation in temperature in the study area can be considered small. In future studies, personal temperature monitoring should be used to obtain more accurate exposure variables. Another limitation was that we could not adjust for the use or availability of air conditioning even though having central air conditioning has been shown to explain some of the disparities in heat-related mortality [44]. It is possible that inability to control for air conditioning lead to weaker than expected associations between high temperatures and inflammation markers. Finally, we did not correct for multiple comparisons in this study; however, we studied inflammation markers that have been associated with cardiovascular outcomes in previous studies [10-12,16-18]. On the other hand, the strengths of our study included the use of a large study cohort with more than 1,200 observations without seasonal restrictions, as in previous studies. We also adjusted the models for a variety of patient characteristics and air pollution as possible confounders, and included time lags up to 4 weeks in order to capture more cumulative effects of temperature.

## Conclusions

We found a linear relationship between decrease in outdoor temperature and the levels of hs CRP, sVCAM-1, and sICAM-1 among elderly men. The associations were acute and cumulative for hs CRP, but mainly 3 to 4 weeks delayed for the adhesion molecules. Our findings suggest that inflammatory markers can be part of the intermediate processes that lead to cardiovascular deaths following exposure to decreasing, but not increasing, temperatures.

## Competing interests

The authors declare that they have no competing interests.

## Authors' contributions

JIH performed the statistical analyses and drafted the manuscript. AZ helped with the statistical analyses and helped to draft the manuscript. DS participated to the organization of the study subjects' clinical visits and critically reviewed the draft. PSV participated in coordinating the clinic visits of the study subjects and reviewed the manuscript. JS conceived and coordinated the study and helped with the statistical analyses and to draft the manuscript. All authors have read and approved the final manuscript.

## Supplementary Material

Additional file 1 **Spearman rank correlations between environmental variables, air pollution and inflammation markers**. Click here for file

Additional file 2 **Associations between temperature and inflammation markers**. The % change (95% confidence intervals) in the levels of interleukins -1β, -6 and -8 for a 5°C decrease in temperature among elderly men. Click here for file

Additional file 3 **Associations between temperature and inflammation markers**. The % change (95% confidence intervals) in the levels of white blood cell count and tumor necrosis factor α for a 5°C decrease in temperature among elderly men. Click here for file

Additional file 4 **The % change in the inflammation markers for a 5°C decrease in ambient temperature**. The % change (95% confidence intervals) in the levels of C-reactive protein, soluble vascular cell adhesion molecule-1, and soluble intercellular adhesion molecule-1 in association with a 5°C decrease in ambient temperature in models adjusted for room temperature. Click here for file
