# Supplementary material for: Associations between outdoor temperature and markers of inflammation: a cohort study
Source: Environ Health. 2010 Jul 23;9:42. doi: 10.1186/1476-069X-9-42 (PMC2920265; doi:10.1186/1476-069X-9-42)
Supplement: Additional file 1 — Spearman rank correlations between environmental variables, air pollution and inflammation markers. [file 1476-069X-9-42-S1.DOC]

**Additional File 1.**

**Table 1.** Spearman rank correlations between environmental variables, air pollution and inflammation markers.

|  | RH | Bp | BC | O_3_ | CRP | IL-1B | IL-6 | IL-8 | TNF | sICAM-1 | sVCAM-1 | WBC^m^ |
| --- | --- | --- | --- | --- | --- | --- | --- | --- | --- | --- | --- | --- |
| Temp^a^ | 0.08 | -0.14 | 0.36 | 0.50 | -0.01 | -0.02 | -0.01 | -0.04 | 0.03 | 0.03 | 0.07 | 0.02 |
| RH^b^ |  | -0.12 | 0.42 | -0.21 | -0.02 | 0.01 | -0.01 | 0.00 | -0.05 | 0.02 | 0.04 | -0.01 |
| Bp^c^ |  |  | 0.13 | -0.23 | -0.01 | -0.02 | -0.02 | -0.01 | -0.03 | -0.02 | -0.03 | -0.02 |
| BC^d^ |  |  |  | -0.15 | -0.02 | 0.00 | -0.04 | -0.02 | -0.03 | 0.01 | 0.09 | 0.02 |
| O_3_^e^ |  |  |  |  | 0.02 | -0.03 | -0.03 | -0.04 | 0.00 | 0.04 | 0.04 | 0.01 |
| CRP^f^ |  |  |  |  |  | 0.04 | 0.07 | 0.05 | 0.10 | 0.22 | 0.05 | 0.20 |
| IL-1β^g^ |  |  |  |  |  |  | 0.53 | 0.54 | 0.44 | 0.06 | 0.03 | 0.00 |
| IL-6^h^ |  |  |  |  |  |  |  | 0.71 | 0.54 | 0.05 | 0.01 | 0.07 |
| IL-8^i^ |  |  |  |  |  |  |  |  | 0.53 | 0.04 | 0.02 | 0.05 |
| TNFα^j^ | |  |  |  |  |  |  |  |  | 0.13 | 0.07 | 0.10 |
| sICAM-1^k^ | |  |  |  |  |  |  |  |  |  | 0.38 | 0.12 |
| sVCAM-1^l^ | |  |  |  |  |  |  |  |  |  |  | 0.08 |

^a^ Ambient temperature, ^b^ Relative humidity, ^c^ Barometric pressure, ^d^ Black carbon, ^e^ Ozone, ^f^ C-reactive protein, ^g^ Interleukin-1beta, ^h^ Interleukin-6, ^i^ Interleukin-8, ^j^ Tumor necrosis factor alpha, ^k^ soluble Intercellular adhesion molecule-1, ^l^ soluble Vascular cell adhesion molecule-1, ^m^ White Blood Cell count
