# Supplementary material for: Associations between outdoor temperature and markers of inflammation: a cohort study
Source: Environ Health. 2010 Jul 23;9:42. doi: 10.1186/1476-069X-9-42 (PMC2920265; doi:10.1186/1476-069X-9-42)
Supplement: Additional file 3 — Associations between temperature and inflammation markers. The % change (95% confidence intervals) in the levels of white blood cell count and tumor necrosis factor α for a 5°C decrease in temperature among elderly men. [file 1476-069X-9-42-S3.DOC]

**Additional File 3.**

**Figure 1. Associations between temperature and inflammation markers.**

The % change (95% Confidence Intervals) in the levels of white blood cell count and tumor necrosis factor α for a 5°C decrease in temperature among elderly men.
