# Supplementary material for: Associations between outdoor temperature and markers of inflammation: a cohort study
Source: Environ Health. 2010 Jul 23;9:42. doi: 10.1186/1476-069X-9-42 (PMC2920265; doi:10.1186/1476-069X-9-42)
Supplement: Additional file 4 — The % change in the inflammation markers for a 5°C decrease in ambient temperature. The % change (95% confidence intervals) in the levels of C-reactive protein, soluble vascular cell adhesion molecule-1, and soluble intercellular adhesion molecule-1 in association with a 5°C decrease in ambient temperature in models adjusted for room temperature. [file 1476-069X-9-42-S4.DOC]

**Additional file 4**

**Table 1. The % change in the inflammation markers for a 5°C decrease in ambient temperature.**

The % change (95% confidence intervals) in the levels of C-reactive protein, soluble vascular cell adhesion molecule-1, and soluble intercellular adhesion molecule-1 in association with decrease in ambient temperature in models adjusted for room temperature.

| Adjusted for Room Temperature | | |
| --- | --- | --- |
|  | % Change | 95% CI |
| C-reactive protein ^a^ |  |  |
| Lag 0 | 8.98 ^*^ | 2.41, 16.0 |
| Lag 1 | 7.15 ^*^ | 0.10, 14.7 |
| Soluble vascular cell adhesion molecule-1 ^a^ | |  |
| Lag 0 | -1.17 | -3.23, 0.94 |
| Lag 1 | 1.23 | -1.07, 3.59 |
| Intercellular cell adhesion molecule-1 ^a^ | |  |
| Lag 0 | 0.67 | -0.70, 2.06 |
| Lag 1 | -0.30 | -1.77, 1.20 |

* *P*-value <0.05

^a^ Model adjusted for room temperature, relative humidity, barometric pressure,

season, time trend,weekday, age, smoking, use of any antihypertensive medication

and statins, body mass index, hypertension, education, diabetes, alcohol use

(≥2 drinks/day), and race
